# Does septic arthritis after anterior cruciate ligament reconstruction lead to poor outcomes? A systematic review and meta-analysis of observational studies

**DOI:** 10.1186/s43019-024-00248-z

**Published:** 2024-12-05

**Authors:** Ashleigh Peng Lin, Bao Tu Thai Nguyen, Son Quang Tran, Yi-Jie Kuo, Shu-Wei Huang, Yu-Pin Chen

**Affiliations:** 1 Department of Medical Education, Taipei Veterans General Hospital, Taipei, Taiwan; 2 The International Ph.D. Program in Medicine, College of Medicine, Taipei Medical University, Taipei, Taiwan; 3 Department of Orthopedics, Faculty of Medicine, Can Tho University of Medicine and Pharmacy, Can Tho, Vietnam; 4 Department of Orthopedics, Wan Fang Hospital, Taipei Medical University, Taipei, Taiwan; 5 Department of Orthopedics, School of Medicine, College of Medicine, Taipei Medical University, Taipei, Taiwan; 6 Department of Applied Science, National Taitung University, Taitung City, Taiwan

**Keywords:** Infection, Septic arthritis, Anterior cruciate ligament reconstruction, Patient-reported outcome, Clinician-reported outcome, Osteoarthritis, Graft retention, Graft removal

## Abstract

**Background:**

Septic arthritis is a rare but devastating complication after anterior cruciate ligament reconstruction (ACLR). While early treatment can prevent significant graft complications, outcomes are often inferior to those in uncomplicated ACLR. Furthermore, whether to retain or remove the graft after infection remains debatable. Therefore, we sought to compare the outcomes of septic arthritis post ACLR with uncomplicated ACLR and evaluate graft retention versus removal in infected patients.

**Methods:**

We conducted a systematic review and meta-analysis in which PubMed, Embase, and Cochrane Library databases were searched. Clinical studies were included if they compared patient-reported, clinician-reported, or radiographic outcomes (minimum follow-up of 12 months) between patients with post-ACLR septic arthritis and those with uncomplicated ACLR or that compared graft retention and removal in patients with post-ACLR septic arthritis.

**Results:**

Thirteen studies were retrieved. Patients with post-ACLR septic arthritis reported inferior Lysholm Knee Scoring Scale scores (mean difference (MD) 7.53; 95% confidence interval (CI) 3.20–11.86; *P* = 0.0006), Tegner Activity Scale scores (MD, 1.42; 95% CI 1.07–1.76; *P* < .00001), and return to sports rates (53% versus 76%, respectively) to those of patients with uncomplicated ACLR. Patients with post-ACLR septic arthritis and those with uncomplicated ACLR did not differ in terms of the pooled estimate of various clinician-reported outcomes, such as the objective International Knee Documentation Committee score, anterior–posterior laxity, pivot shift, and Lachman test results. Furthermore, no significant difference was noted between the aforementioned patient groups regarding osteoarthritis (detected radiographically). Graft retention led to better patient- and clinician-reported outcomes than graft removal.

**Conclusions:**

Despite similar clinician-reported outcomes and osteoarthritis rates, patients with post-ACLR septic arthritis reported worse outcomes than those with uncomplicated ACLR. Graft retention leads to improved patient- and clinician-reported outcomes compared with the outcomes of graft removal. Our findings may help develop realistic expectations and management strategies for this rare complication.

**Supplementary Information:**

The online version contains supplementary material available at 10.1186/s43019-024-00248-z.

## Introduction

Septic arthritis after anterior cruciate ligament (ACL) reconstruction (ACLR) is a rare but devastating complication, with an incidence rate of 0.14–1.8% [1]. Although prompt infection control can improve functional outcomes without graft laxity or retears, the outcomes of post-ACLR septic arthritis are often inferior to those of uncomplicated ACLR [2]. Poor knee function may substantially affect patients, preventing them from attaining their goals and ultimately leading to dissatisfaction.

Current management strategies involving early surgical debridement and concomitant intravenous antibiotic therapy minimize the severity of inflammation, thus preventing articular cartilage degradation [3–5]. However, whether successful infection eradication and graft recovery translates into clinical, patient-reported, and radiographic outcomes similar to those of patients with uncomplicated ACLRs at mid- to long-term follow-up remains debatable. Although some studies have reported inferior subjective and objective outcomes, such as functional knee scores, sports and activity levels, joint laxity, and radiographic osteoarthritis [6–9], others have reported similar outcomes [10]. Furthermore, outcomes may vary depending on graft retention or removal. Graft removal minimizes the risk of persistent infection, but ACL deficiency may increase the risk of additional meniscal and cartilage damage. By contrast, graft retention provides adequate stability but may lead to further damage and instability with persistent infection [11].

Very few studies have comprehensively compared post-ACLR septic arthritis and uncomplicated ACLR in terms of their outcomes. Small sample sizes limit the power of most studies, resulting in unreliable findings. As most affected patients are young and active athletes, understanding the prognosis of post-ACLR infection in this population is crucial. Because of the rarity of post-ACLR septic arthritis, clinical data may be required to optimize the clinical management and counseling of affected patients. Therefore, we conducted this systematic review and meta-analysis of the clinical, functional, and radiographic outcomes of septic arthritis developing at least 12 months after ACLR. Our objective was to examine whether there were differences in outcomes between patients with post-ACLR septic arthritis and those without complications. Additionally, we sought to determine whether outcomes varied between patients who retained their graft and those who required graft removal due to post-ACLR septic arthritis. We hypothesized that patients developing septic arthritis following ACLR would have both subjective and objective outcomes that were inferior compared with those without complications. Furthermore, we anticipated that graft removal in post-ACLR septic arthritis would result in worse outcomes.

## Methods

### Study design

This systematic review and meta-analysis was conducted in accordance with the Preferred Reporting Items for Systematic Reviews and Meta-Analyses (PRISMA) guidelines [12]. This study was registered in the PROSPERO online public database (CRD42023390990).

### Search strategy

To identify relevant studies, we searched the PubMed, Embase, and Cochrane Library databases from the inception of the databases up to January 2023. We used the following broad search terms: *anterior cruciate ligament reconstruction* AND (*septic arthritis* OR *infection*). Search terms were mapped to Medical Subject Headings terms where possible. All relevant references were checked for additional and unpublished citations. Afterward, all articles were combined into a single list, and duplicates were removed.

### Selection criteria

We included studies comparing patients with post-ACLR septic arthritis with those without it in terms of outcomes. In addition, we included studies comparing the outcomes of graft retention with those of graft removal in patients with post-ACLR septic arthritis. Other inclusion criteria were as follows: availability of age and sex data of patients with post-ACLR septic arthritis and those with uncomplicated ACLR; follow-up period of at least 12 months, comparison of at least one outcome of interest between patients with post-ACLR septic arthritis and those with uncomplicated ACLR or between graft retention and graft removal in patients with post-ACLR septic arthritis, availability of data regarding treatment protocols for patients with post-ACLR septic arthritis, and publication in English-language peer-reviewed journals. The articles included in this review study met all of the aforementioned criteria. No restrictions were imposed for index ACLR type (primary or revision), graft choice, participant matching method, or cartilage or meniscus treatment method.

We reviewed the abstracts and excluded animal studies, commentaries or opinion pieces, review articles reporting data presented in already identified articles, and articles presenting primary data duplicated in another included article. In the case of duplicate data, we selected the articles with the most complete baseline information concerning the post-ACLR septic arthritis and uncomplicated ACLR groups and the graft retention and removal groups. After exclusion, a second reviewer reviewed the remaining studies for subsequent meta-analysis.

### Data extraction

Data regarding patient characteristics, such as age, sex, and follow-up duration, were extracted to obtain an overview of the population. The diagnostic criteria for post-ACLR septic arthritis used in each study were also extracted. Surgical data, such as ACLR type (primary or revision), graft used for the index ACLR, prior knee procedures, and concomitant meniscal and cartilage surgery, were extracted (if reported) to compare the septic arthritis and uncomplicated ACLR groups. In addition, information regarding the management of septic arthritis was extracted. To compare the graft retention and removal groups, data regarding infection management, including time to presentation, Gächter stage, total number of irrigation and debridement (I&D), and graft reimplantation, were extracted (if reported). Two reviewers worked independently: one extracted the relevant data from the included studies, and another verified the extracted data. Any discrepancies between the two reviewers were resolved through consensus or by a third reviewer.

### Methodological quality appraisal

We judged the quality of the included studies by assessing various aspects of study design that would likely introduce bias, such as variables prone to measurement bias, insufficient adjustment for confounding factors, and loss to follow-up for observational studies. To compare the septic arthritis and uncomplicated ACLR groups, we evaluated pre-exposure, at-exposure, post-exposure, and overall biases by using the Risk of Bias in Nonrandomized Studies of Exposures (ROBINS-E) tool [13]. To compare the graft retention and removal groups, we evaluated pre-intervention, at-intervention, post-intervention, and overall biases using the Risk of Bias in Nonrandomized Studies of Interventions (ROBINS-I) tool [14].

### Outcomes

The main outcome measures were patient-reported outcomes, clinician-reported outcomes, and osteoarthritis risk. Patient-reported outcomes included Lysholm Knee Scoring Scale score, Tegner Activity Scale score, Knee Injury and Osteoarthritis Outcome Score (KOOS), subjective International Knee Documentation Committee (IKDC) score, and return to sports rate. Clinician-reported outcomes included objective IKDC score, KT-1000 score, pivot shift test result, and Lachman test result; the objective IKDC results were analyzed in terms of the number of knees classified as abnormal/severely abnormal (IKDC category C or D), and the pivot shift and Lachman test results were analyzed in terms of the number of patients with a grade of at least 1. Osteoarthritis was assessed on the basis of radiographic grading, and osteoarthritis was classified according to the grading used in each article. Data regarding the number of patients in each group with radiographic evidence of osteoarthritis were obtained.

### Statistical analysis

Outcomes were pooled for meta-analysis by using RevMan (version 5.4.0) [14]. Continuous variables are presented as mean ± standard deviation (SD) values. If SD values were not reported, we contacted the corresponding authors and requested the statistical data. When authors could not be contacted, we calculated SD values using the available data according to a previously reported validated formula [15]. The mean difference (MD) and 95% confidence interval (CI) values were calculated for dichotomous variables. A random-effects model (DerSimonian and Laird) was used to compute the pooled estimates [16]. Cochrane *Q* tests and *I*^2^ statistics were used to evaluate the statistical heterogeneity and inconsistency among the effects of the included studies, respectively. Statistical significance was set at *P* < 0.05 for the Cochrane *Q* tests. Statistical heterogeneity was assessed using the* I*^2^ test, with *I*^2^ quantifying the proportion of the total outcome variability attributable to the variability among the studies. In addition, subgroup analyses were performed by pooling the estimates for similar patient subsets among studies, as appropriate.

## Results

### Included studies

**Fig. 1:**
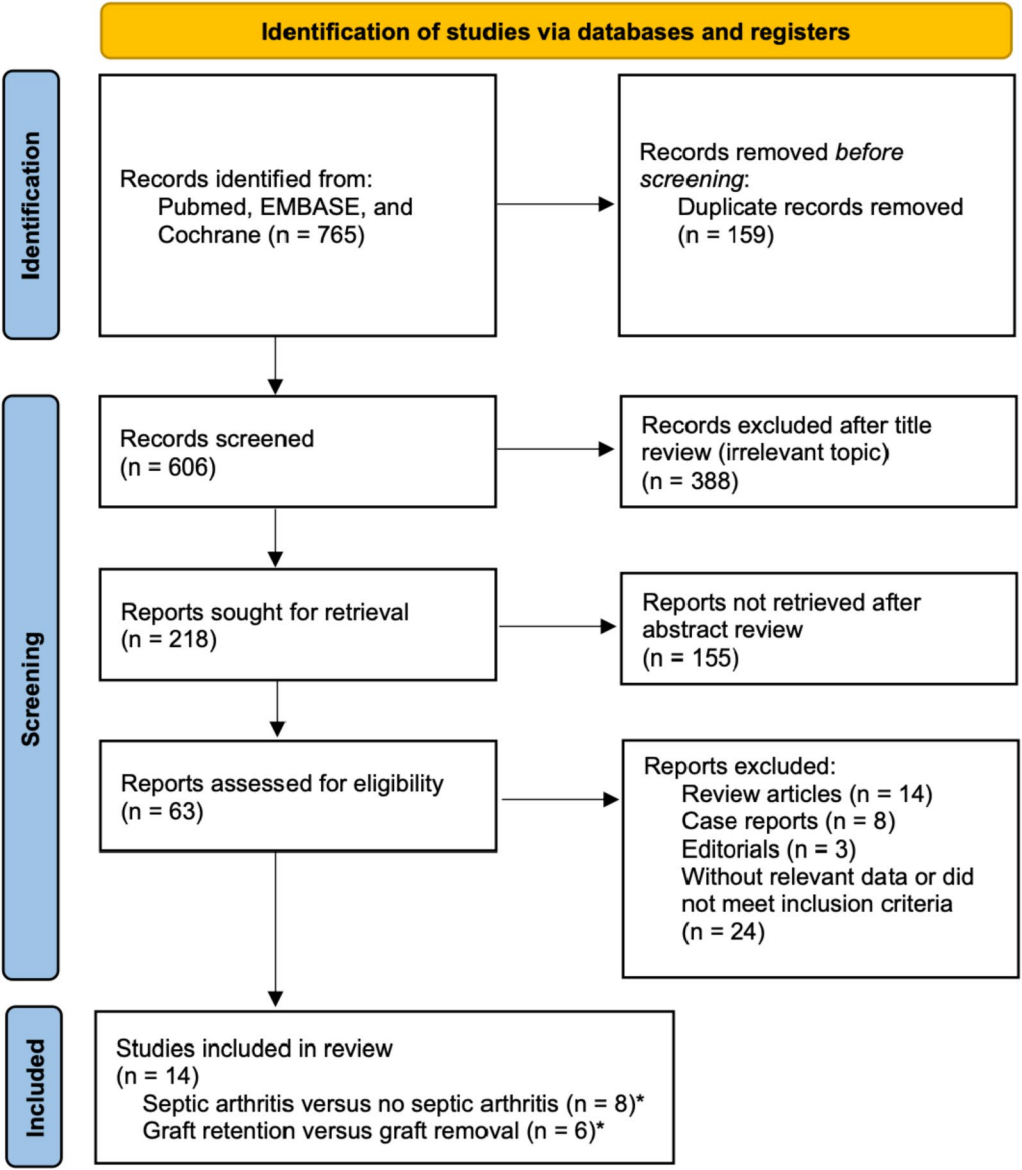
PRISMA flowchart for article selection. *One study was included in both subgroups

The searches yielded 2425 entries. After removing duplicates and excluding irrelevant articles, 183 were independently reviewed by two reviewers. Finally, 13 studies met the inclusion criteria and were selected for analysis (Fig. 1). Most of the studies (10 out of 13) reported the criteria of septic arthritis diagnosis, which included a combination of history, physical examination, and synovial fluid cultures (Supplementary Table 1).

### Results of risk of bias assessment

The quality of evidence varied between the outcomes. High-quality evidence was rare (Supplementary Tables 2 and 3). Most studies were uncontrolled cohort studies or case series. If a single study was published in several outlets, we analyzed only the study with the most complete dataset to avoid duplication. Studies were divided into two categories, each corresponding to a research question of the present meta-analysis.

### Demographic characteristics

#### Post-ACLR septic arthritis versus uncomplicated ACLR

Eight studies reported the clinical, functional, or radiographic outcomes of post-ACLR septic arthritis versus uncomplicated ACLR (Table 1) [7, 17–23]. A total of 6773 patients (septic arthritis group, 129; uncomplicated ACLR group, 6644) were included in this study. Patient demographics were generally similar between the septic arthritis and uncomplicated ACLR groups. The mean age was 29.0 ± 8.4 and 28.5 ± 10.8 years in the septic arthritis and uncomplicated ACLR groups, respectively (*P* = 0.59), and 72.1% and 71.5% of the respective groups were men (*P* = 0.44). The percentage of primary ACLR was also similar between the two groups (93.8% versus 94.2%; *P* = 0.43). However, the percentage of prior knee procedure (32.0% versus 7.3%; *P* < 0.05) and concomitant surgery (28.6% versus 46.7%; *P* < 0.05) was different between the two groups.

**Table 1:** Characteristics of studies comparing patients with post-ACLR septic arthritis and those without it

No.	Study [year]	Study design Country	LOE	Study characteristics	No. of patients	Age (years), mean ± SD	Male, *n* (%)	Primary ACLR, *n* (%)	Graft type, *n* (%)			Prior knee procedure, *n* (%)	Concomitant surgery, *n* (%)	Follow-up period (months), mean ± SD (range)
									BPTB	Hamstring	Allograft			
1	Abdel-Aziz [17]	Prospective Egypt	II	Patients with ACLR with hamstring autograft; 2004–2011; CU	S: 24 N: 24	S: 26 ± 5 N: 27 ± 4	S: 24 (100) N: 24 (100)	NR	S: 0 (0) N: 0 (0)	S: 24 (100) N: 24 (100)	S: 0 (0) N: 0 (0)	S: 4 (17) N: NR	NR	S: 59 ± 21 (18–96) N: 55 ± 22 (18–96)
2	Bohu [18]	Prospective France	III	Patients with ACLR; 2012–2016; CdS	S: 7 N: 1802	S: 36.4 ± 13.7 N: 29.1 ± 9.7	S: 6 (86) N: 1626 (90)	S: 5 (71) N: 1627 (90)	S: 2 (29) N: 172 (0.1)	S: 5 (71) N: 1520 (84)	S: 0 (0) N: 0 (0)	S: 4 (57) N: 210 (12)	694 (38)^b^ 61 (3)^c^ 376 (21)^d^ 355 (20)^e^	33.6 ± 14.4 (18.0–57.6)
3	Boström Windhamre [19]	Retrospective Sweden	III	Patients with primary ACLR; 2001–2009; CAC with complete rehabilitation	S: 27 N: 27	S: 27 (16–43)^*^ N: 28 (14–43)^*^	S: 13 (48) N: 13 (48)	S: 27 (100) N: 27 (100)	S: 0 (0) N: 0 (0)	S: 27 (100) N: 27 (100)	S: 0 (0) N: 0 (0)	S: 11 (41) N: 12 (44)	S: 10 (37)^f^ N: 9 (33)^f^	S: 60 (13–108)^*^ N: 66 (16–114)^*^
4	Brophy [20]	Prospective United States	IV	Patients with ACLR; 2002–2008; MOON	S: 21 N: 3189	S: 25.8 ± 11.3 N: 26.6 ± 11.0	S: 12 (57) N: 1778 (56)	S: 18 (86) N: 2982 (94)	S: 4 (19) N: 1401 (44)	S: 12 (57) N: 1093 (34)	S: 5 (24) N: 695 (22)	NR	NR	S: 78 ± 3.6 N: 78 ± 4.8
5	Calvo [21]	Retrospective Chile	IV	Patients with primary ACLR with hamstring autograft; 2000–2011; CA	S: 7 N: 1557	S: 27.8 (14–51)^*^ N: 28.3 (14–55)^*^	S: 7 (100) N: 1281 (82)	S: 7 (100) N: 1557 (100)	S: 0 (0) N: 0 (0)	S: 7 (100) N: 1557 (100)	S: 0 (0) N: 0 (0)	S: 0 (0) N: 20 (1)	S: 3 (42)^f^ N: 745 (48)^a,f,g^	18–108^†^
6	Meglic [22]	Prospective Slovenia	II	Patients with primary ACLR; 2004–2014; UMCL with complete rehabilitation	S: 18 N: 20	S: 31 ± 7 N: 33 ± 6	S: 11 (61) N: 12 (60)	S: 18 (100) N: 20 (100)	S: 11 (61) N: 12 (60)	S: 7 (39) N: 8 (40)	S: 0 (0) N: 0 (0)	NR	S: 2 (11)^a^ N: 2 (10)^a^	S: 48 ± 4 (42–56) N: 48 ± 4 (42–56)
7	Schollin-Borg [7]	Retrospective, Sweden	III	Patients with ACLR; 1996–1999; UUH	S: 10 N: 10	S: 28.3 ± 5.5 N: 29.1 ± 5.7	S: 8 (80) N: 8 (80)	NR	S: 6 (60) N: 6 (60)	S: 4 (40) N: 3 (30)	S: 0 (0) N: 0 (0)	S: 5 (50) N: 7 (70)	S: 4 (40)^f,g^ N: 2 (20)^f^	36^**^
8	Torres-Claramunt [23]	Retrospective, Spain	IV	Patients with ACLR; 2006–2009; PdSM and ICATME with rehabilitation	S: 15 N: 15	S: 33.5 ± 7.6 N: 34.7 ± 7.6	S: 12 (76) N: 10 (67)	NR	S: 4 (27) N: 5 (33)	S: 11 (73) N: 10 (67)	S: 0 (0) N: 0 (0)	NR	S: 3 (20)^f^ N: NR	S: 39.3 ± 13 N: 42.6 ± 7.5

^*^Mean (range), ^﻿†^Range, and ^**^Mean

^a^Meniscal repair

^b^Extra-articular tenodesis (tensor fasciae latae)

^c^Chondroplasty

^d^Partial medial meniscectomy and suturing

^e^Partial lateral meniscectomy and suturing

^f^Meniscectomy

^g^Meniscus microfracture

*ACLR* anterior cruciate ligament reconstruction, *BPTB* bone–patellar tendon–bone graft, *CA* Clinica Alemana, *CAC* Capio Artro Clinic, *CdS* Clinique du Sport, *CU* Cairo University, *ICATME* ICATME-Institut Universitari Dexeus, *LOE* level of evidence, *MOON* Multicenter Orthopaedic Outcomes Network knee group, *N* patients without post-ACLR septic arthritis, *NR* not reported, *PdSM* Parc de Salut Mar, *S* patients with septic arthritis, *SD* standard deviation, *UMCL* University Medical Centre Ljublijana, *UUH* Uppsala University Hospital

#### Graft retention versus graft removal in patients with post-ACLR septic arthritis

Six studies compared clinical or functional outcomes between the graft retention and removal groups (Supplementary Table 3) [1, 8, 11, 21, 24, 25]. After the removal of duplicates, 91 patients (graft retention group, 56; graft removal, 35) were included in this study. In general, patient demographics were similar between the two groups. The mean age was 28.8 ± 7.8 and 29.3 ± 9.7 years in the graft retention and removal groups, respectively (*P* = 0.80), and 89.6% and 78.3% of the respective groups were men (*P* = 0.20). The percentage of index primary ACLR (83.7% versus 76.2%; *P* = 0.46) and hamstring autograft were also similar between the two groups (69.6% versus 62.9%; *P* = 0.50). The mean follow-up period was 45.89 months.

### Septic arthritis presentation and treatment protocol

The mean time to infection presentation and treatment is presented in Supplementary Table 5. In all eight studies, septic arthritis was managed with arthroscopic I&D and antibiotic treatment [7, 17–23]. The number of irrigation procedures ranged from 1 to 11. Graft retention (rate, 71–100%) was reported in all eight studies, whereas subsequent surgery (rate, 0–39%) was reported in five studies (Supplementary Table 5) [17, 18, 20, 22].

### Patient-reported outcomes

#### Lysholm Knee Scoring Scale scores

**Fig. 2:**
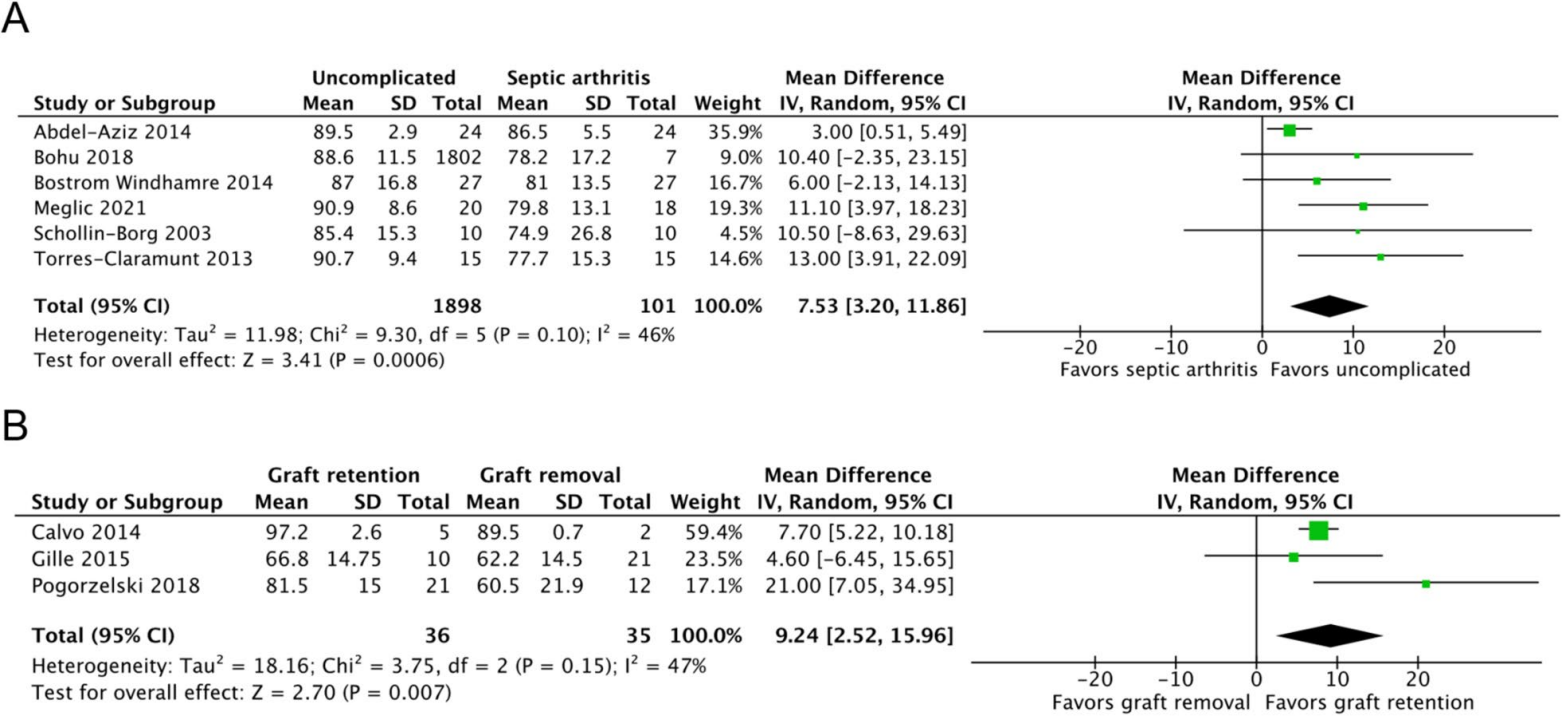
Lysholm Knee Scoring Scale scores of the (**A**) septic arthritis and uncomplicated ACLR groups and the (**B**) graft retention and removal groups. An inverse-variance random-effects model was used for meta-analysis. Mean differences are presented in terms of 95% confidence interval values

Six studies, including 1999 patients, reported the Lysholm Knee Scoring Scale scores of septic arthritis (*n* = 101) and uncomplicated ACLR (*n* = 1898) groups [7, 17–19, 22, 23]. The mean timepoint for assessment was at 35.36 months. The septic arthritis group had significantly lower scores than the uncomplicated ACLR group (MD, 7.53; 95% CI 3.20–11.86; *P* = 0.0006; *I*^2^, 46%; *P* = 0.10; Fig. 2A).

Three studies, including 71 patients, reported the Lysholm Knee Scoring Scale scores of the graft retention (*n* = 36) and removal (*n* = 35) groups [11, 21, 24]. The graft retention group reported significantly higher scores than did the graft removal group at a mean follow-up of 57.47 months (MD, 9.24; 95% CI 2.52–15.96; *P* = 0.007; *I*^2^, 47%; *P* = 0.15; Fig. 2B).

#### Tegner activity scale scores

**Fig. 3:**
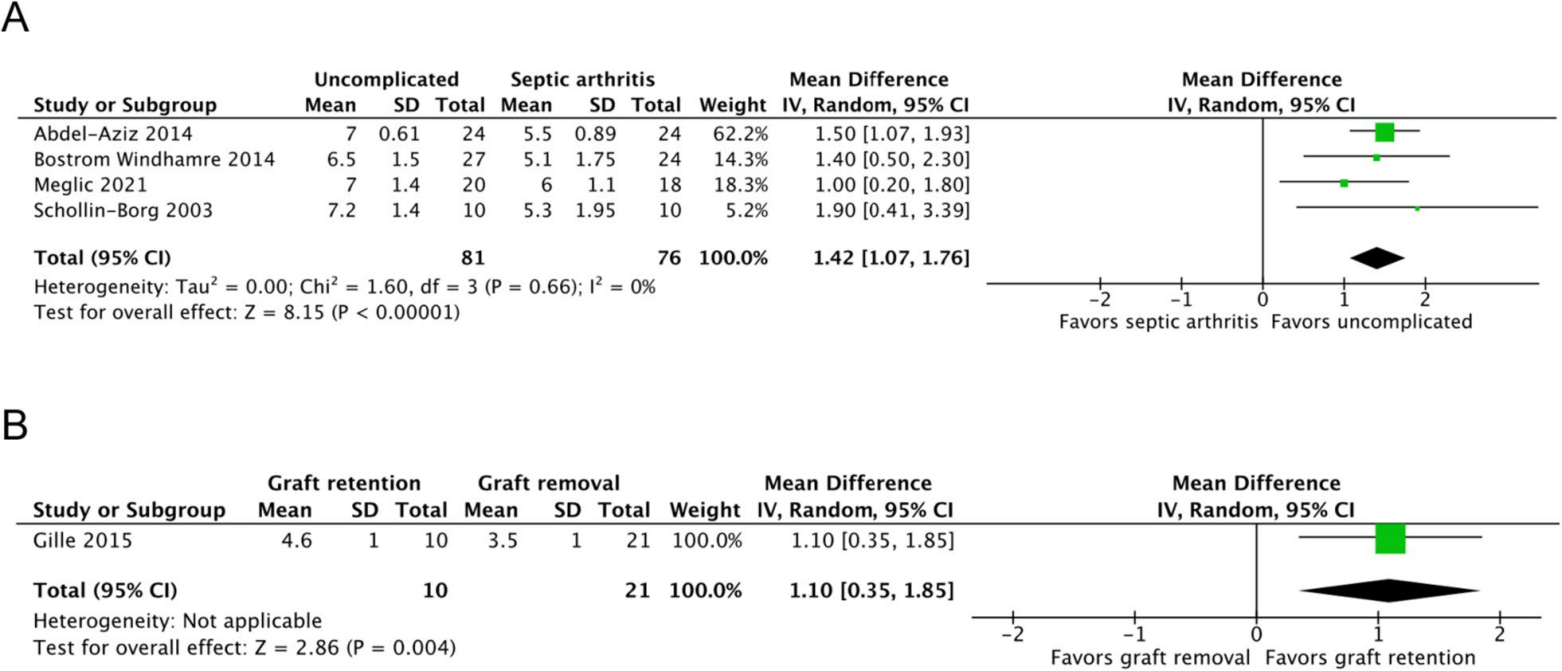
Tegner Activity Scale scores of the (**A**) septic arthritis and uncomplicated ACLR groups and the (**B**) graft retention and removal groups. An inverse-variance random-effects model was used for meta-analysis. Mean differences are presented in terms of 95% confidence interval values

Four studies, including 157 patients, reported the Tegner Activity Scale scores of septic arthritis (*n* = 76) and uncomplicated ACLR (*n* = 81) groups [7, 17, 19, 22]. The septic arthritis group had significantly lower scores than did the uncomplicated ACLR group at a mean follow-up of 54.26 months (MD, 1.42; 95% CI 1.07–1.76; *P* < 0.00001; *I*^2^, 0%; *P* = 0.66; Fig. 3A).

One study reported the Tegner Activity Scale scores of the graft retention (*n* = 10) and removal (*n* = 21) groups, respectively [24]. The graft retention group had higher scores than the graft removal group at 71 months of follow-up (MD, 1.10; 95% CI 0.35–1.85; *P* = 0.004).

#### KOOS

Five studies, including 4689 patients, reported the KOOS of septic arthritis (*n* = 83) and uncomplicated ACLR (*n* = 4606) groups [7, 17–20]. The overall KOOS varied significantly between the two groups at 62.04 months of follow-up (MD, 8.88; 95% CI 3.27–14.49; *P* = 0.002; *I*^2^, 96%; *P* ≤ 0.00001). As depicted in Supplementary Fig. 1, the pooled mean difference estimates were significant for the domains of symptoms (MD, 6.88; 95% CI 1.76–12.00; *P* = 0.008; *I*^2^, 61%; *P* = 0.05), pain (MD, 6.34; 95% CI 3.10–9.58; *P* = 0.0001; *I*^2^, 61%; *P* = 0.05), sports and recreation (MD, 9.37; 95% CI 2.11–16.64; *P* = 0.01; *I*^2^, 62%; *P* = 0.05), and quality of life (MD, 11.84; 95% CI 3.26–20.43; *P* = 0.007; *I*^2^, 73%; *P* = 0.01) at 62.14 months of follow-up. However, the results corresponding to the activities of daily living (ADL) were not significant (MD, 2.99; 95% CI −1.34–7.32; *P* = 0.18; *I*^2^, 77%; *P* = 0.004).

#### Subjective IKDC scores

Three studies, including 4597 patients, reported the subjective IKDC scores of septic arthritis (*n* = 37) and uncomplicated ACLR (4560) groups [18, 20, 23]. The septic arthritis group reported significantly lower subjective IKDC scores than did the uncomplicated group at 62.05 months of follow-up (MD, 10.45; 95% CI 2.00–18.90; *P* = 0.02; *I*^2^, 81%; *P* = 0.005; Supplementary Fig. 2A).

One study, including 33 patients, reported the subjective IKDC scores of the graft retention (*n* = 21) and removal (*n* = 12) groups [11]. The graft retention group had significantly higher scores than the graft removal group at 49.27 months of follow-up (MD, 21.00; 95% CI 7.05–34.95; *P* = 0.003; Supplementary Fig. 2B).

#### Return to sports rates

**Fig. 4:**
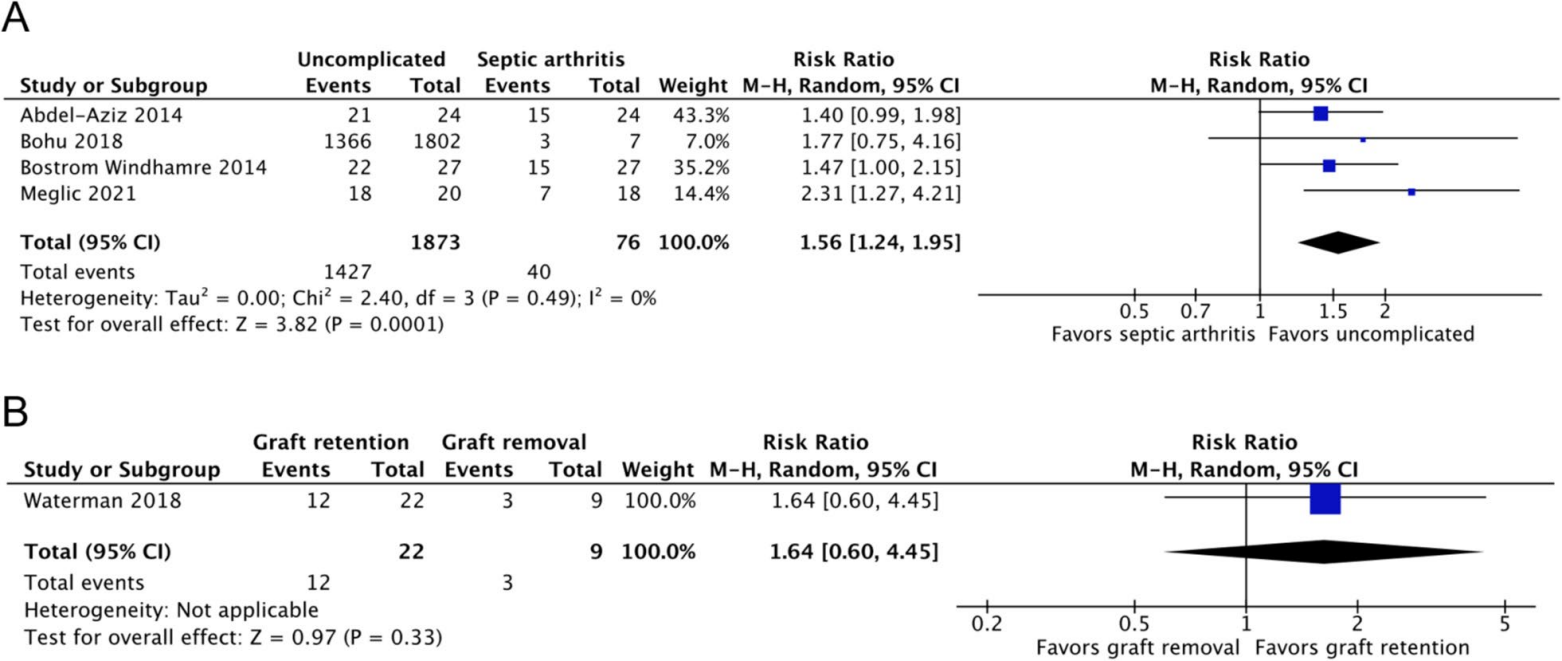
Return to sports rates of the (**A**) septic arthritis and uncomplicated ACLR groups and the (**B**) graft retention and removal groups. A Mantel–Haenszel random-effects model was used for meta-analysis. Risk ratios are presented in terms of 95% confidence interval values

Four studies, including 1949 patients, reported the return to sports rates of septic arthritis (*n* = 76) and uncomplicated ACLR (*n* = 1873) groups [17–19, 22]. Compared with 76% of patients with uncomplicated ACLR, 53% of patients with post-ACLR septic arthritis returned to sports by the end of follow-up (risk ratio [RR], 1.56; 95% CI 1.24–1.95; *P* = 0.0001; *I*^2^, 0%; *P* = 0.49; Fig. 4A). The mean timepoint for assessment was at 35.27 months of follow-up.

One study, including 31 patients, reported the return to sports rates of the graft retention (*n* = 22) and removal (*n* = 9) groups [1]. No significant difference was noted between the two groups at the mean of 29.6 months of follow-up (RR, 1.64; 95% CI 0.60–4.45; *P* = 0.33; Fig. 4B).

### Clinician-reported outcomes

#### Objective IKDC scores

**Fig. 5:**
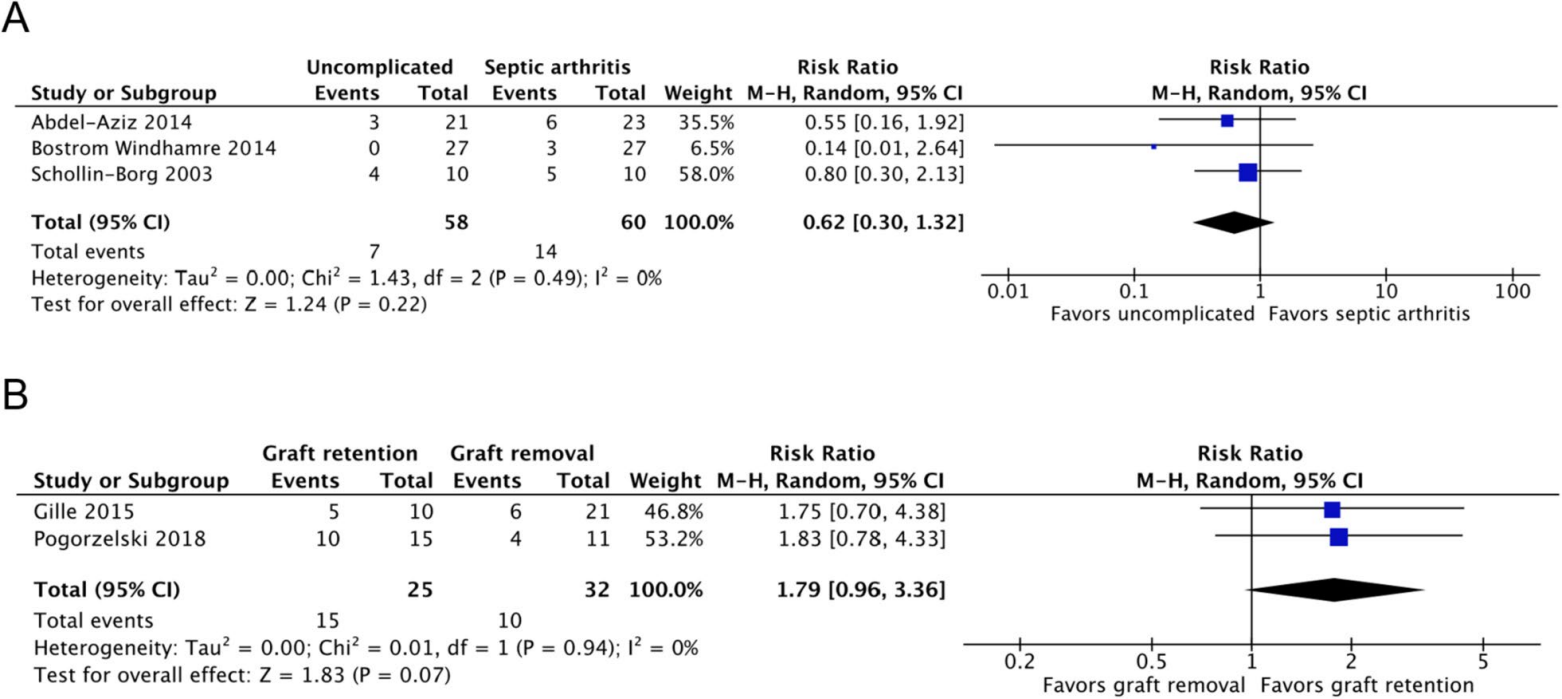
Objective IKDC scores of the (**A**) septic arthritis and uncomplicated ACLR groups and the (**B**) graft retention and removal groups. A Mantel–Haenszel random-effects model was used for meta-analysis. Risk ratios are presented in terms of 95% confidence interval values

Three studies, including 118 patients, reported the objective IKDC scores of septic arthritis (*n* = 60) and uncomplicated ACLR (*n* = 58) groups [7, 17, 19]. No significant difference was observed between the two groups (RR, 0.62; 95% CI 0.30–1.32; *P* = 0.22; *I*^2^, 0%; *P* = 0.49; Fig. 5A). The mean timepoint for assessment was at 56.21 months of follow-up.

Two studies, including 57 patients, reported the objective IKDC scores of the graft retention (*n* = 25) and graft removal (*n* = 32) groups [11, 24]. No significant difference was noted between the two groups at 57.47 months of follow-up (MD, 1.79, 95% CI 0.96–3.36; *P* = 0.07; *I*^2^, 0%; *P* = 0.94; Fig. 5B).

#### Anterior–posterior laxity side-to-side differences

**Fig. 6:**
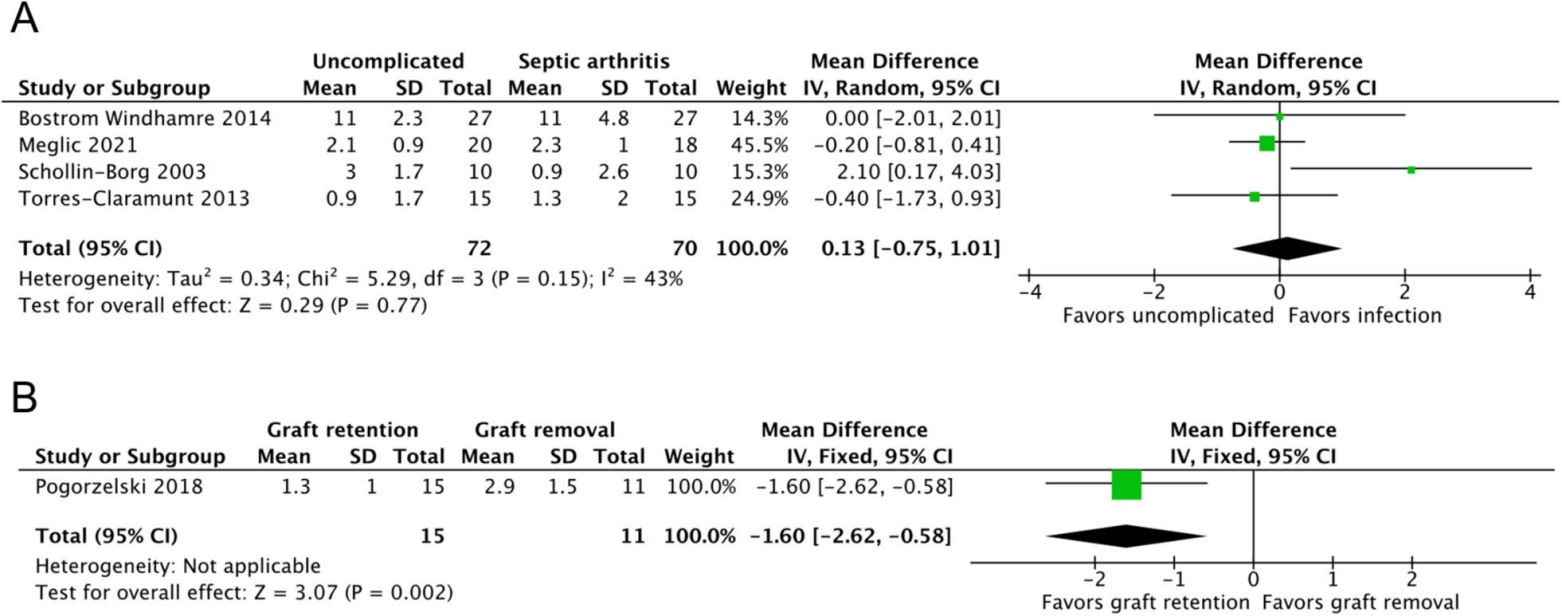
KT-1000 scores of the (**A**) septic arthritis and uncomplicated ACLR groups and the (**B**) graft retention and removal groups. An inverse-variance random-effects model was used for meta-analysis. Mean differences are presented in terms of 95% confidence interval values

Four studies, including 142 patients, reported the mean KT-1000 scores of septic arthritis (*n* = 70) and uncomplicated ACLR (*n* = 72) groups [7, 19, 22, 23]. No significant difference was noted between the two groups (MD, 0.13; 95% CI 0.75–1.01; *P* = 0.77; *I*^2^, 43%; *P* = 0.15; Fig. 6A). The mean timepoint for assessment was at 50.52 months of follow-up.

One study including 26 patients (graft retention group, 15; graft removal group, 11) reported significantly less laxity in the graft retention group compared with the graft removal group at 49.27 months of follow-up (MD, −1.60; 95% CI −2.62 to −0.58; *P* = 0.0002; Fig. 6B) [11].

#### Pivot shift and Lachman test results

Two studies, including 80 patients, reported the pivot shift and Lachman test results of septic arthritis (*n* = 37) and uncomplicated ACLR (*n* = 43) groups [17, 22]. No significant difference was observed in the risk of positive results between the two groups at the mean of 53.02 months of follow-up (pivot shift test: RR, 0.75; 95% CI 0.36–1.58; *P* = 0.45; *I*^2^, 0%; *P* = 0.83; Lachman test: RR, 0.63; 95% CI 0.24–1.62; *P* = 0.34; *I*^2^, 0%; *P* = 0.66; Supplementary Fig. 3A, B).

### Radiographic osteoarthritis

**Fig. 7:**
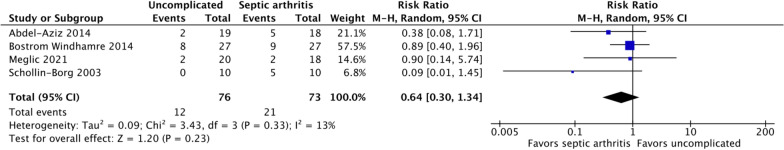
Radiographic osteoarthritis risks of the septic arthritis and uncomplicated ACLR groups. A Mantel–Haenszel random-effects model was used for meta-analysis. Risk ratios are presented in terms of 95% confidence interval values

Radiographic evaluation for osteoarthritis was reported in four studies, including 149 patients (septic arthritis group, 73; uncomplicated ACLR group, 76) [7, 17, 19, 22]. Radiographic evidence of osteoarthritis was noted in 29% and 16% of all patients in septic arthritis and uncomplicated ACLR groups, respectively. No significant difference was observed in the risk of osteoarthritis between the two groups (RR, 0.64; 95% CI 0.30–1.34; *P* = 0.23; *I*^2^, 13%; *P* = 0.33; Fig. 7). The mean timepoint for radiographic assessment was 54.26 months of follow-up.

## Discussion

Our meta-analysis indicated that patients with post-ACLR septic arthritis reported poor outcomes at a follow-up of at least 12 months. However, the objective outcomes of post-ACLR septic arthritis, including clinician-reported outcomes and radiographic osteoarthritis, were not inferior to those of uncomplicated ACLR. Graft retention led to better patient- and clinician-reported outcomes than graft removal.

Because of the rarity of post-ACLR septic arthritis, large-scale studies of outcomes after treatment are limited in number; therefore, the synthesis of clinical data is crucial for obtaining evidence for clinicians to set realistic expectations regarding the clinical, functional, and radiographic outcomes of this complication. Compared with other reviews [10, 26–28], this study offered an in-depth review that evaluated the outcomes of patients who develop septic arthritis following ACLR and sought to determine whether graft retention or removal was the more effective treatment for these cases.

In this study, the subjective outcomes of post-ACLR septic arthritis were inferior to those of uncomplicated ACLR. Subjective knee functionality, measured based on the Lysholm Knee Scoring Scale and IKDC subjective scores, was significantly lower in the septic arthritis group than in the uncomplicated ACLR group. The average Lysholm Knee Scoring Scale score of the septic arthritis group was 7.5 points less than that of the uncomplicated ACLR group. Similarly, the average subjective IKDC score of the septic arthritis group was 10.45 points less than that of the uncomplicated ACLR group. To the best of our knowledge, no consensus has been achieved on the minimal clinically important differences (MCID) between the IKDC subjective scores; nevertheless, a 10-point difference may be clinically important, with MCID of 8.7 and 9.0, reported by Nwachukwu et al.’s studies, respectively [29, 30]. Scores corresponding to most KOOS dimensions were significantly lower in the septic arthritis group than in the uncomplicated ACLR group. Notably, KOOS and ADL were similar between the two groups, which is consistent with the trend noted in other studies—ADL is not strongly affected after ACLR [31–33]. In general, these findings suggest that, after septic arthritis, patients perceive their knee function to be inferior to that of patients with uncomplicated ACLR but adequate for ADL.

The objective outcomes of post-ACLR septic arthritis were similar to those of uncomplicated ACLR. The clinician-reported evaluation of knee status, which was assessed on the basis of objective IKDC scores, revealed no significant difference between the two groups in the risk of clinical failure. Both static and rotational joint laxity, assessed through the KT-1000 or Lachman and pivot shift test, were similar between the groups. These findings are quite similar to those reported in Torus-Claramunt et al.’s study, which also suggested that, if graft could be retained after the treatment of septic arthritis, the laxity obtained could be similar to that in patients who have not suffered an infection [23].

Counter to our expectation, the risk of osteoarthritis, as detected through radiography, was comparable between the two groups at a mean of 54.26 months of follow-up. Early I&D and antibiotic treatment in most studies may explain similar objective outcomes [34]. Because radiographs may not be as sensitive as magnetic resonance imaging (MRI) in distinguishing damage to cartilage surfaces and surrounding soft tissue, the early signs of osteoarthritis might have been missed [22, 35].

Despite similar knee laxity between the groups, the septic arthritis group had lower activity levels, as assessed using the Tegner Activity Scale, and return to sports rates compared with the uncomplicated group. The discrepancy between solid clinical outcome parameters and compromised activity and return to sports rates may be attributed to postoperative psychological dysfunction. Psychological factors, such as fear of reinjury and self-efficacy, have been reported as reasons for not returning to physical functioning [36–38]. Although this aspect has largely been highlighted in patients with uncomplicated ACLR, those with post-ACLR septic arthritis who undergo additional surgery and rehabilitation may perceive their condition as more severe than that of patients with uncomplicated ACLR; this perception may exacerbate their fear of returning to sports and activity [25]. Addressing these psychosocial factors may have implications for rehabilitation because they may influence the collaborative functional goals set by clinicians and patients.

Although consensus has been achieved on using early surgical intervention and intravenous antibiotics in patients with post-ACLR infection, whether to retain or remove the grafts remains debatable. Studies supporting graft removal have highlighted the increased risk of persistent infection, reoperation, and functional ACL deficiency with retention, whereas other studies have reported good clinical results after graft retention [2, 27, 39–42]. Graft retention appears to be associated with improved patient- and clinician-reported outcomes compared with the outcomes of graft removal. These results are consistent with those of a previous systematic review of 19 studies, including 203 patients, which found consistently better subjective and objective clinical outcomes reported by studies with higher rates of graft retention [10]. In general, these superior outcomes empirically confirm what one would intuitively expect: graft retention minimizes anatomic disruption and morbidity and rehabilitation from additional reconstructive surgery, all of which may affect clinical, functional, and patient-reported outcomes. However, it is important to note that it may not always be appropriate for a clinician to decide to retain a graft if a patient continues to show persistent infection with graft retention or if the graft shows significant structural damage. At that point, even if graft retention shows more favorable outcomes, clinical circumstances may require graft removal.

The present study has some limitations. First, throughout the review, the level of evidence was of a relatively low grade because few high-quality studies included in our review compared post-ACLR septic arthritis with uncomplicated ACLR or graft retention with removal in patients with post-ACLR septic arthritis; this limited our interpretation and conclusions. Second, although we attempted to match patients’ demographic characteristics, some confounding factors, including index ACLR type, graft type, prior or concomitant surgeries, and severity of infection, were not necessarily matched between the groups. Additionally, although limited range of motion is a significant complication of septic arthritis, we were unable to analyze this outcome variable owing to the diversely different presentation among the included studies. Finally, osteoarthritis was detected through plain radiography rather than by MRI, which could have detected the early signs of osteoarthritis. Furthermore, the number of patients in each cohort assessed for osteoarthritis was few, which may not be significant enough for the power of the study. Despite these limitations, the findings should remain of substantial interest to clinicians because they expand the evidence on the mid- to long-term outcomes of post-ACLR septic arthritis.

## Conclusions

Despite similar clinician-reported outcomes and osteoarthritis rates, patients with post-ACLR septic arthritis reported worse outcomes than those with uncomplicated ACLR. Graft retention leads to improved patient- and clinician-reported outcomes compared with the outcomes of graft removal. Our findings may help develop realistic expectations and management strategies for this rare complication.

## Supplementary Information

Additional file 1

## Data Availability

All data analyzed in this study are included in these published articles [1, 7, 8, 11, 17–25].

## Abbreviations

ACL: Anterior cruciate ligament
ACLR: Anterior cruciate ligament reconstruction
PRISMA: The Preferred Reporting Items for Systematic Reviews and Meta-Analyses
I&D: Irrigation and debridement
ROBINS-E: The Risk of Bias in Nonrandomized Studies of Exposures
ROBINS-I: The Risk of Bias in Nonrandomized Studies of Interventions
KOOS: Knee Injury and Osteoarthritis Outcome Score
IKDC: International Knee Documentation Committee
SD: Standard deviation
CI: Confidence interval
RR: Risk ratio
ADL: Activities of daily living
MRI: Magnetic resonance imaging
