# Supplementary material for: Does septic arthritis after anterior cruciate ligament reconstruction lead to poor outcomes? A systematic review and meta-analysis of observational studies
Source: Knee Surg Relat Res. 2024 Dec 5;36:45. doi: 10.1186/s43019-024-00248-z (PMC11622541; doi:10.1186/s43019-024-00248-z)
Supplement: Supplementary file 1 — Additional file 1 [file 43019_2024_248_MOESM1_ESM.docx]

| **Supplementary Table 1**. Definition of septic arthritis after anterior cruciate ligament reconstruction | | |
| --- | --- | --- |
| **No** | **Study [Year]** | **Definition** |
| 1 | Abdel-Aziz [2014]^17^ | A positive culture from a knee aspiration or a cell count consistent with intraarticular infection (over 10,000 cells/μl polymorphonuclear cells) in the synovial fluid in patients presenting with symptoms consistent with septic arthritis. |
| **2** | Bohu [2018]^18^ | The appearance of clinical signs of septic arthritis (heat, pain, redness, local knee swelling, and/or local knee joint drainage) within 3 months after reconstruction. The diagnosis was confirmed under bacteriology from deep tissue samples obtained during revision surgery. |
| 3 | Boström Windhamre [2014]^19^ | Not specified |
| 4 | Brophy [2019]^20^ | Cases with a postoperative infection occurred that required surgical irrigation and debridement were determined “infection” ones. |
| 5 | Calvo [2014]^21^ | The diagnosis of intraarticular infection was made in patients with altered inflammation parameters and an alteration of joint fluid analysis. |
| 6 | Gille [2014]^24^ | Not specified |
| 7 | Meglic [2021]^22^ | Positive history and knee examination  Elevated serum inflammatory markers (C-reactive protein > 5mg/L and erythrocyte sedimentation rate > 28mm/h), and joint aspiration with synovial white blood cell count > 40,000 cells/mm^3^ and positive Gram stain. |
| 8 | Pogorzelski [2018]^11^ | Criteria 1: History, Examination, Elevated C-reactive protein, Elevated leukocytes  Criteria 2: Positive intra-articular biopsy cultures |
| 9 | Schollin Borg [2003]^7^ | Clinical symptoms, laboratory studies, and joint fluid culture results but not specified |
| 10 | Schulz [2007]^8^ | Not specified |
| 11 | Torres-Claramunt [2013]^23^ | Either a positive culture from a knee aspiration or a suggestive blood laboratory test (systemic white blood cell count and C-reactive protein) in patients with septic arthritis symptoms. |
| 12 | Themessl [2022]^25^ | Criteria 1: History, Examination, Elevated C-reactive protein, Elevated leukocytes  Criteria 2: Positive intra-articular biopsy cultures |
| 13 | Waterman [2018]^1^ | The presence of a synovial white blood cell counts over 50,000, frank purulence, and/or positive synovial fluid cultures after knee arthrocentesis. |

| **Supplementary Table 2.** Assessment of Methodological Quality of the Included Studies (ROBINS-E) | | | | | | | | | | | |
| --- | --- | --- | --- | --- | --- | --- | --- | --- | --- | --- | --- |
| **No.** | **Author [year]** | **Pre-exposure** | | | | **Post-exposure** | | | | **Overall bias** |  |
|  |  | **Bias due to confounding** | **Bias from measurement of the exposure** | **Selection bias** | **Bias due to post-exposure interventions** | | **Bias due to missing data (attrition)** | **Bias in measurement of outcomes** | **Bias in selection of reported results** |  |  |
| 1 | Abdel-Aziz [2014]^17^ | Moderate | Low | Low | Low | | Moderate | Moderate | Low | Moderate |  |
| 2 | Bohu [2018]^18^ | Moderate | Low | Low | Low | | Moderate | Low | Low | Moderate |  |
| 3 | Brophy [2019]^20^ | Serious | Moderate | Moderate | Moderate | | Moderate | Moderate | Low | Serious |  |
| 4 | Calvo [2014]^21^ | Moderate | Low | Low | Low | | Low | Moderate | Low | Moderate |  |
| 5 | Meglic [2021]^22^ | Moderate | Low | Low | Moderate | | Moderate | Low | Low | Moderate |  |
| 6 | Schollin-Borg [2003]^7^ | Moderate | Low | Low | Low | | Low | Low | Low | Moderate |  |
| 7 | Torres-Claramunt [2013]^23^ | Moderate | Low | Moderate | Low | | Low | Low | Low | Moderate |  |
| 8 | Windhamre [2014]^19^ | Moderate | Low | Low | Moderate | | Low | Low | Low | Moderate |  |

| **Supplementary Table 3.** Assessment of Methodological Quality of the Included Studies (ROBINS-I) | | | | | | | | | |
| --- | --- | --- | --- | --- | --- | --- | --- | --- | --- |
| **No.** | **Author [year]** | **Pre-intervention** | | **At-intervention** | | **Post-intervention** | | | **Overall bias** |
|  |  | **Bias due to confounding** | **Selection bias** | **Bias in classification of interventions** | **Deviation from intended interventions** | **Bias due to missing data (attribution)** | **Bias in measurement of outcomes** | **Bias in selection of reported results** |  |
| 1 | Gille [2014]^24^ | Serious | Low | Low | Moderate | Moderate | Moderate | Low | Serious |
| 2 | Pogorzelski [2018]^11^ | Low | Low | Low | Low | Moderate | Moderate | Low | Moderate |
| 3 | Schulz [2007]^8^ | Serious | Low | Low | Moderate | Moderate | Moderate | Low | Serious |
| 4 | Themessl [2022]^25^ | Low | Low | Low | Low | Moderate | Moderate | Low | Moderate |
| 5 | Waterman [2018]^1^ | Moderate | Low | Low | Low | Low | Moderate | Moderate | Moderate |

| **Supplementary Table 4** Characteristics of studies comparing graft retention and graft removal in post-ACLR septic arthritis patients | | | | | | | | | | | | | | |
| --- | --- | --- | --- | --- | --- | --- | --- | --- | --- | --- | --- | --- | --- | --- |
| No. | Study [Year] | Study design, country | LOE | Study characteristics | No. of patients | Age, y  Mean ± SD | Male sex  n (%) | Index ACLR primary  n (%) | Hamstring autograft  n (%) | Time to presentation, day  Mean (range) | Gaechter stage, Median (range) | Total I&D  Median (range) | Graft reimplantation  n | Follow-up, month  Mean (range) |
| 1 | Calvo [2014]^21^ | Retrospective, Chile | IV | Post-ACLR septic arthritis managed with arthroscopic I&D 2000-2011 at CA | T: 5  M: 2 | T: 25.8 ± 14.8  M: 33 ± 4.2 | T: 5 (100)  M: 2 (100) | NR | T: 5 (100)  M: 2 (100) | T: 5.4  (4-7)  M: 23.5  (17-30) | NR | T: 1 (1)  M: 3 (3) | NR | 18-108^†^ |
| 2 | Gille [2014]^24^; Schulz [2007]^8^ | Prospective, Germany | III | Post-ACLR septic arthritis managed with arthroscopic treatment or arthrotomy 1993- 2010 at UKSH | T: 8  M: 12 | 34.7 (17-49)^*^ | NR | NR | 9 (29) | 78 (3-243) | Stage 2: 10  Stage 3: 18  Stage 4: 3 | 2.6  (2-5) | 2 of 12 | 71  (13-140) |
| 3 | Pogorzelski [2018]^11^; Themessl [2022]^25^ | Retrospective, Germany | III | Post-ACLR septic arthritis managed with arthroscopic I&D 2006- 2015 at HRdI | T: 21  M: 12 | T: 29.7 ± 7.9  M: 27.5 ± 12.7 | T: 18 (85.7)  M: 8 (66.7) | T: 14 (67)  M: 7 (58) | T: 20 (95.2)  M: 12 (100) | T: 15.5  (6-95)^**^  M: 23  (6-90)^**^ | T: 2 (1-2)  M: 2 (1-3) | T: 3  (1-7)  M: 4  (2-5) | 4 of 12 | T: 54  (18-103)  M: 41  (13-86) |
| 4 | Waterman [2018]^1^ | Retrospective, US | IV | Primary ACLR with secondary septic arthritis managed with arthroscopic I&D 2007-2013 in USMHS | T: 22  M: 9 | 26.8 ± 4.5 | 28 (90.3) | T: 22 (100)  M: 9 (100) | 17 (55) | 55.1 (5-477) | NR | 2.3  (1-4) | 7 of 9 | 26.9 |
| *Indicates mean (range), **indicates median (range), †indicates range  Abbreviations: ACLR, anterior cruciate ligament reconstruction; CA, Clinica Alemana; HRdI, Hospital Rechts der Isar; I&D, irrigation and debridement; LOE, level of evidence; M, graft removal group; SD, standard deviation; T, graft retention group; UKSH, University of Schleswig-Holstein hospital; USMHS, US Military Health System | | | | | | | | | | | | | | |

| S**upplementary Table 5.** Details of management and outcomes of septic arthritis after ACLR | | | | | | | | |  |
| --- | --- | --- | --- | --- | --- | --- | --- | --- | --- |
| No. | Study [Year] | Time, day,  Mean ± SD (range) | | Treatment protocol | No. of irrigation procedures  Mean (range) | Graft retention  n (%) | Subsequent surgery  n (%) |  |  |
|  |  | Initial ACLR to infection symptoms | Infection symptoms to treatment |  |  |  |  |  |  |
| 1 | Abdel-Aziz [2014]^17^ | 12.4 ± 8.8  (5-45) | NR | Arthroscopic I&D and synovectomy with graft retention and cephalosporin or vancomycin for 21-42 days (IV) | 2.8 (1-6) | 22 (92) | 4 (17)^c^ |  |  |
| 2 | Bohu [2018]^18^ | 15.7 ± 5.5  (7-21) | < 1.0 | Arthroscopic I&D with graft retention and vancomycin and tazocilline for 6 weeks (IV followed by oral) | 1.4 (1-2) | 7 (100) | 0 |  |  |
| 3 | Boström Windhamre [2014]^19^ | 8 (1-22)^*^ | 4.8 (0-16)^*^ | Arthroscopic I&D and cloxacillin or clindamycin (10 days IV followed by 7.6 weeks oral) | 3.7 (1-11) | 27 (100) | NR |  |  |
| 4 | Brophy [2019]^20^ | 39 ± 47^a^ | Arthroscopic or open I&D and antibiotic treatment | NR | 17 (80) | 8 (38)^b^ |  |  |  |
| 5 | Calvo [2014]^21^ | 10.6 ± 9.6  (4-30) | 0^d^ | Arthroscopic I&D and broad-spectrum antibiotics for 6 weeks (IV followed by oral) | 1.6 (1-3) | 5 (71) | NR |  |  |
| 6 | Meglic [2021]^22^ | 11.7 ± 2.4 | 1.4 ± 0.7 | Arthroscopic I&D with graft retention and antibiotic treatment for 6 weeks | 1.2 (1-3) | 18 (100) | 0 |  |  |
| 7 | Schollin-Borg [2003]^7^ | 9.5 ± 4.8  (4-20) | NR | Arthroscopic I&D and antibiotic treatment for 4-12 weeks (IV cloxacillin followed by oral flucloxacillin) | NR | 10 (100) | 4 (40)^e^ |  |  |
| 8 | Torres-Claramunt [2013]^23^ | 23.9 ± 14^a^ | | Arthroscopic I&D, ceftazidime and vancomycin for 6 weeks (IV followed by oral) | 1.3 (1-3) | 14 (93) | NR |  |  |
| *Indicates mean (range)  ^a^Time between index ACLR and surgical I&D  ^b^Includes three revision ACLRs and one total knee arthroplasty  ^c^Three graft ruptures (one revision ACLR, other two refused) and three arthroscopic partial meniscectomies following another injury  ^d^At time of clinical diagnosis  ^e^Includes two partial meniscal excisions, one arthroscopic lysis of adhesions, and one secondary ACLR (graft rupture)  Abbreviations: ACLR, anterior cruciate ligament reconstruction; I&D, irrigation and debridement; IV, intravenous; SD, standard deviation | | | | | | | | | |


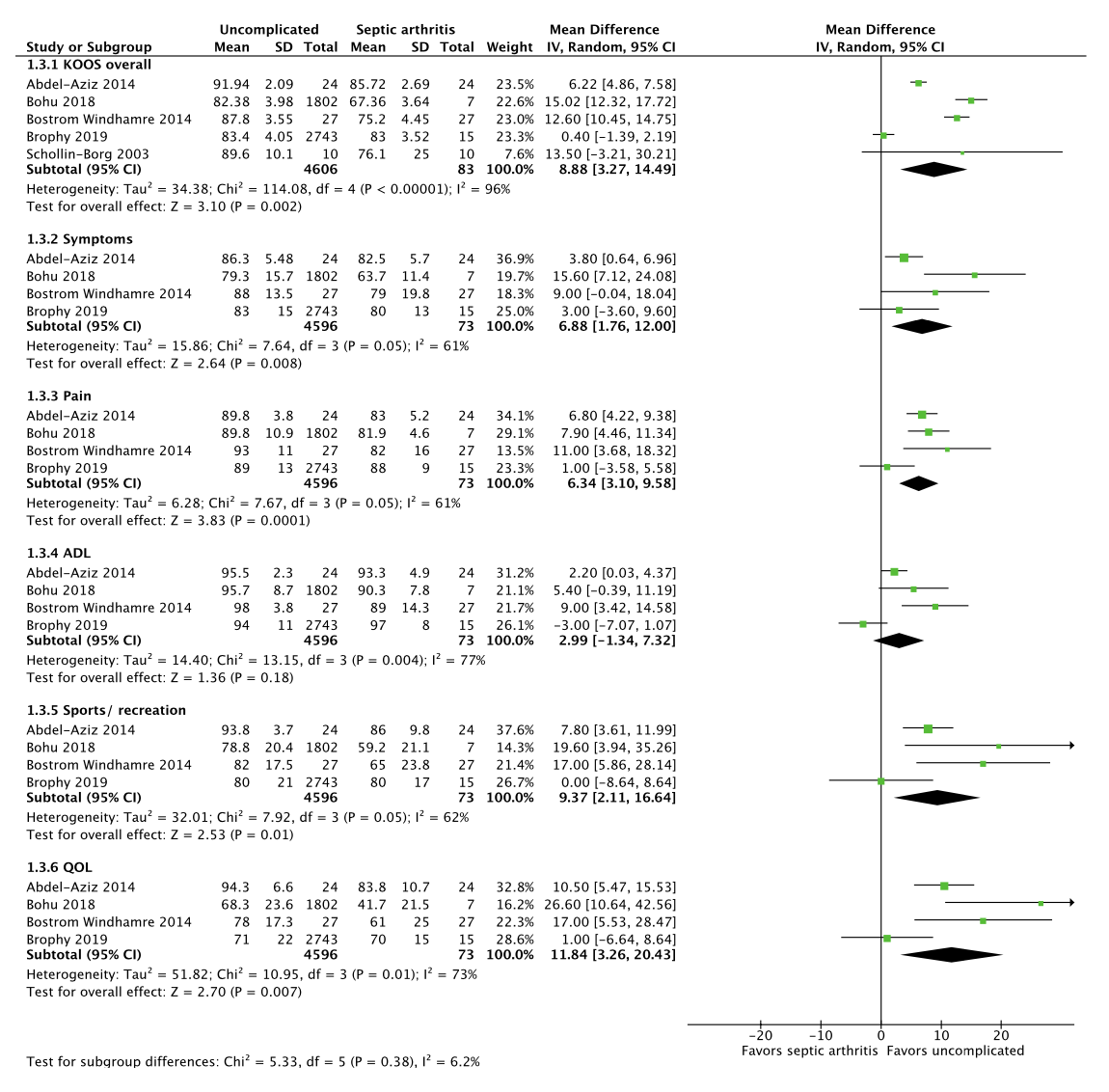


**Supplementary Figure 1.** KOOS scores of septic arthritis versus uncomplicated ACLR. An inverse variance random-effects model was used for meta-analysis. Mean difference are shown with 95 per cent confidence intervals.

A)


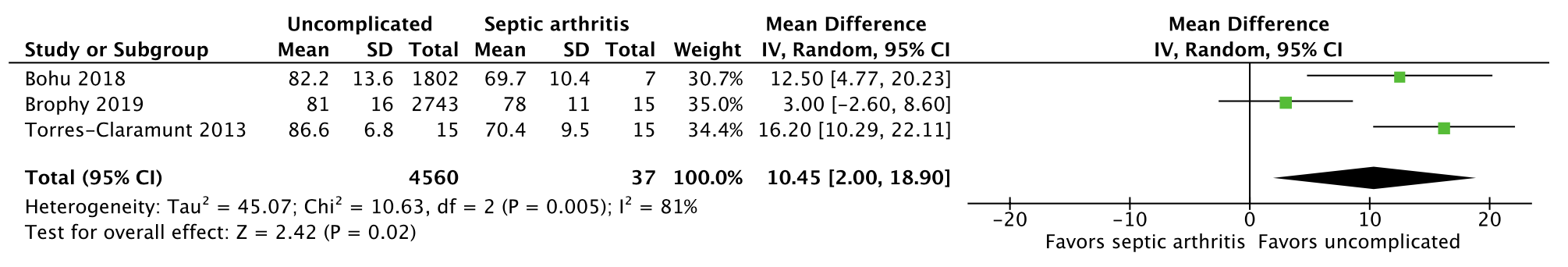


B)


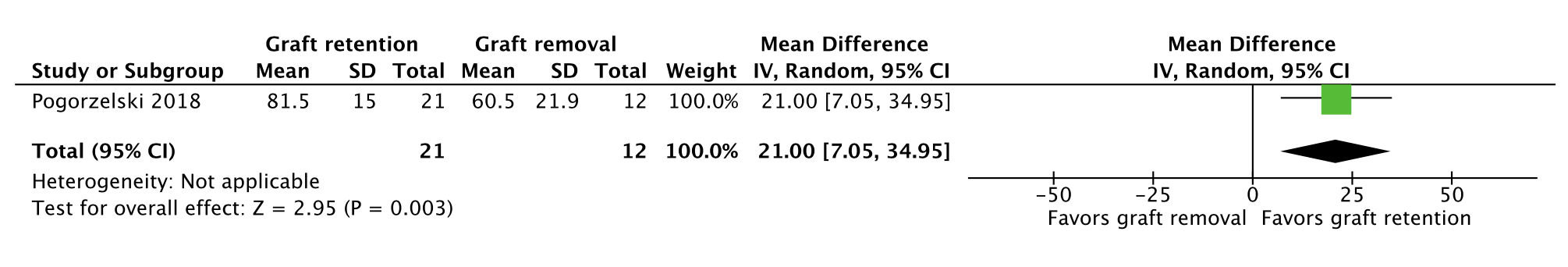


**Supplementary Figure 2.** Subjective IKDC scores of A) septic arthritis versus uncomplicated ACLR and B) graft retention versus graft removal. An inverse variance random-effects model was used for meta-analysis. Mean difference are shown with 95 per cent confidence intervals.

A)


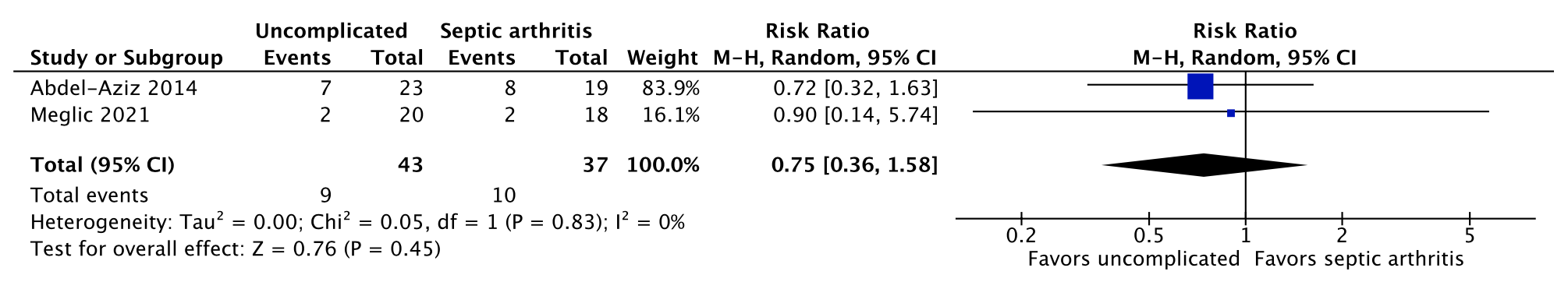


B)


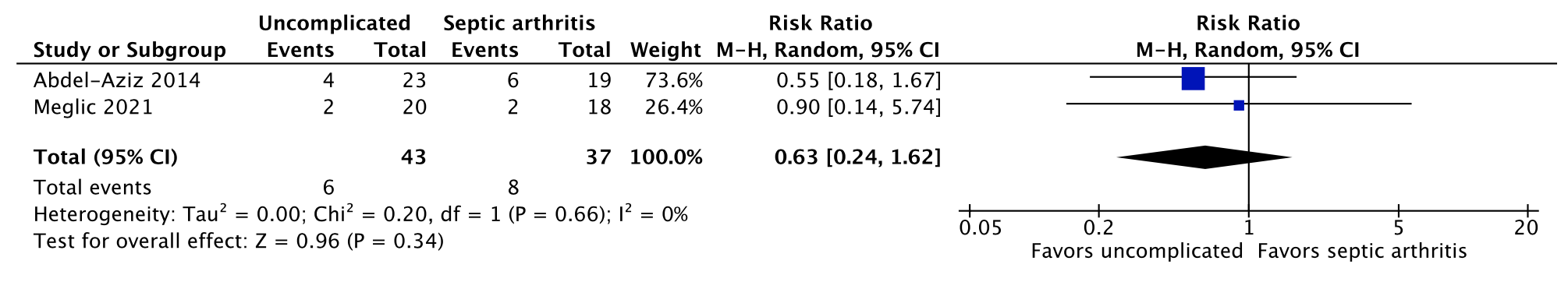


**Supplementary Figure 3.** A) Pivot shift test and B) Lachman test of septic arthritis versus uncomplicated ACLR. A Mantel-Haenszel random-effects model was used for meta-analysis. Risk ratios are shown with 95 per cent confidence intervals.

| **Section and Topic** | **Item #** | **Checklist item** | **Location where item is reported** |
| --- | --- | --- | --- |
| **TITLE** | | |  |
| Title | 1 | Identify the report as a systematic review. | Title page |
| **ABSTRACT** | | |  |
| Abstract | 2 | See the PRISMA 2020 for Abstracts checklist. | Manuscript page 1 |
| **INTRODUCTION** | | |  |
| Rationale | 3 | Describe the rationale for the review in the context of existing knowledge. | Manuscript page 3 |
| Objectives | 4 | Provide an explicit statement of the objective(s) or question(s) the review addresses. | Manuscript page 4 |
| **METHODS** | | |  |
| Eligibility criteria | 5 | Specify the inclusion and exclusion criteria for the review and how studies were grouped for the syntheses. | Manuscript page 5 |
| Information sources | 6 | Specify all databases, registers, websites, organisations, reference lists and other sources searched or consulted to identify studies. Specify the date when each source was last searched or consulted. | Manuscript page 4-5 |
| Search strategy | 7 | Present the full search strategies for all databases, registers and websites, including any filters and limits used. | Manuscript page 4-5 |
| Selection process | 8 | Specify the methods used to decide whether a study met the inclusion criteria of the review, including how many reviewers screened each record and each report retrieved, whether they worked independently, and if applicable, details of automation tools used in the process. | Manuscript page 5 |
| Data collection process | 9 | Specify the methods used to collect data from reports, including how many reviewers collected data from each report, whether they worked independently, any processes for obtaining or confirming data from study investigators, and if applicable, details of automation tools used in the process. | Manuscript page 6 |
| Data items | 10a | List and define all outcomes for which data were sought. Specify whether all results that were compatible with each outcome domain in each study were sought (e.g. for all measures, time points, analyses), and if not, the methods used to decide which results to collect. | Manuscript page 6-7 |
|  | 10b | List and define all other variables for which data were sought (e.g. participant and intervention characteristics, funding sources). Describe any assumptions made about any missing or unclear information. | Manuscript page 6-7 |
| Study risk of bias assessment | 11 | Specify the methods used to assess risk of bias in the included studies, including details of the tool(s) used, how many reviewers assessed each study and whether they worked independently, and if applicable, details of automation tools used in the process. | Manuscript page 6 |
| Effect measures | 12 | Specify for each outcome the effect measure(s) (e.g. risk ratio, mean difference) used in the synthesis or presentation of results. | Manuscript page 7-8 |
| Synthesis methods | 13a | Describe the processes used to decide which studies were eligible for each synthesis (e.g. tabulating the study intervention characteristics and comparing against the planned groups for each synthesis (item #5)). | Manuscript page 7-8 |
|  | 13b | Describe any methods required to prepare the data for presentation or synthesis, such as handling of missing summary statistics, or data conversions. | Manuscript page 7-8 |
|  | 13c | Describe any methods used to tabulate or visually display results of individual studies and syntheses. | Manuscript page 7-8 |
|  | 13d | Describe any methods used to synthesize results and provide a rationale for the choice(s). If meta-analysis was performed, describe the model(s), method(s) to identify the presence and extent of statistical heterogeneity, and software package(s) used. | Manuscript page 7-8 |
|  | 13e | Describe any methods used to explore possible causes of heterogeneity among study results (e.g. subgroup analysis, meta-regression). | Manuscript page 7-8 |
|  | 13f | Describe any sensitivity analyses conducted to assess robustness of the synthesized results. | Manuscript page 7-8 |
| Reporting bias assessment | 14 | Describe any methods used to assess risk of bias due to missing results in a synthesis (arising from reporting biases). | Manuscript page 6 |
| Certainty assessment | 15 | Describe any methods used to assess certainty (or confidence) in the body of evidence for an outcome. | Manuscript page 7-8 |
| **RESULTS** | | |  |
| Study selection | 16a | Describe the results of the search and selection process, from the number of records identified in the search to the number of studies included in the review, ideally using a flow diagram. | Manuscript page 8-9 |
|  | 16b | Cite studies that might appear to meet the inclusion criteria, but which were excluded, and explain why they were excluded. | Manuscript page 8-9 |
| Study characteristics | 17 | Cite each included study and present its characteristics. | Manuscript page 10 |
| Risk of bias in studies | 18 | Present assessments of risk of bias for each included study. | Manuscript page 9-10 |
| Results of individual studies | 19 | For all outcomes, present, for each study: (a) summary statistics for each group (where appropriate) and (b) an effect estimate and its precision (e.g. confidence/credible interval), ideally using structured tables or plots. | Manuscript page 11-15 |
| Results of syntheses | 20a | For each synthesis, briefly summarise the characteristics and risk of bias among contributing studies. | Manuscript page 11-15 |
|  | 20b | Present results of all statistical syntheses conducted. If meta-analysis was done, present for each the summary estimate and its precision (e.g. confidence/credible interval) and measures of statistical heterogeneity. If comparing groups, describe the direction of the effect. | Manuscript page 11-15 |
|  | 20c | Present results of all investigations of possible causes of heterogeneity among study results. | Manuscript page 11-15 |
|  | 20d | Present results of all sensitivity analyses conducted to assess the robustness of the synthesized results. | Manuscript page 11-15 |
| Reporting biases | 21 | Present assessments of risk of bias due to missing results (arising from reporting biases) for each synthesis assessed. | Manuscript page 11-15 |
| Certainty of evidence | 22 | Present assessments of certainty (or confidence) in the body of evidence for each outcome assessed. | Manuscript page 11-15 |
| **DISCUSSION** | | |  |
| Discussion | 23a | Provide a general interpretation of the results in the context of other evidence. | Manuscript page 15-18 |
|  | 23b | Discuss any limitations of the evidence included in the review. | Manuscript page 15-18 |
|  | 23c | Discuss any limitations of the review processes used. | Manuscript page 15-18 |
|  | 23d | Discuss implications of the results for practice, policy, and future research. | Manuscript page 18 |
| **OTHER INFORMATION** | | |  |
| Registration and protocol | 24a | Provide registration information for the review, including register name and registration number, or state that the review was not registered. | Manuscript page 4 |
|  | 24b | Indicate where the review protocol can be accessed, or state that a protocol was not prepared. | Manuscript page 4 |
|  | 24c | Describe and explain any amendments to information provided at registration or in the protocol. | N/A |
| Support | 25 | Describe sources of financial or non-financial support for the review, and the role of the funders or sponsors in the review. | Title page |
| Competing interests | 26 | Declare any competing interests of review authors. | Title page |
| Availability of data, code and other materials | 27 | Report which of the following are publicly available and where they can be found: template data collection forms; data extracted from included studies; data used for all analyses; analytic code; any other materials used in the review. | Manuscript |

*From:*  Page MJ, McKenzie JE, Bossuyt PM, Boutron I, Hoffmann TC, Mulrow CD, et al. The PRISMA 2020 statement: an updated guideline for reporting systematic reviews. BMJ 2021;372:n71. doi: 10.1136/bmj.n71

For more information, visit: <http://www.prisma-statement.org/>
